# Integrated Family Planning and Immunization Service Delivery at Health Facility and Community Sites in Dowa and Ntchisi Districts of Malawi: A Mixed Methods Process Evaluation

**DOI:** 10.3390/ijerph17124530

**Published:** 2020-06-24

**Authors:** Chelsea M. Cooper, Jacqueline Wille, Steven Shire, Sheila Makoko, Asnakew Tsega, Anne Schuster, Hannah Hausi, Hannah Gibson, Hannah Tappis

**Affiliations:** 1 Maternal and Child Survival Program, Jhpiego, Washington, DC 20036, USA; jacqueline.wille@jhpiego.org (J.W.); anne.cb.schuster@gmail.com (A.S.); 2 Maternal and Child Survival Program, Jhpiego, Lilongwe, Malawi; steven.shire@jhpiego.org (S.S.); sheilamakoko@gmail.com (S.M.); hannah.gibson@jhpiego.org (H.G.); 3 Maternal and Child Survival Program, John Snow, Inc., Arlington, VA 22202, USA; asnakewy@gmail.com; 4 Maternal and Child Survival Program, John Snow, Inc., Lilongwe, Malawi; hhausi@mw.jsi.com

**Keywords:** Malawi, family planning, contraception, immunization, reproductive health, child health, integrated service delivery, evaluation

## Abstract

The Government of Malawi’s *Health Sector Strategic Plan II* highlights the importance of service integration; however, in practice, this has not been fully realized. We conducted a mixed methods evaluation of efforts to systematically implement integrated family planning and immunization services in all health facilities and associated community sites in Ntchisi and Dowa districts during June 2016–September 2017. Methods included secondary analysis of service statistics (pre- and postintervention), focus group discussions with mothers and fathers of children under age one, and in-depth interviews with service providers, supervisors, and managers. Results indicate statistically significant increases in family planning users and shifts in use of family planning services from health facilities to community sites. The intervention had no effect on immunization doses administered or dropout rates. According to mothers and fathers, benefits of service integration included time savings, convenience, and improved understanding of services. Provision and use of integrated services were affected by availability of human resources and commodities, community linkages, data collection procedures and availability, sociocultural barriers, organization of services, and supervision and commitment of health surveillance assistants. The integration approach was perceived to be feasible and beneficial by clients and providers.

## 1. Introduction

A woman’s access to family planning services and consequential ability to space or limit her pregnancies has a profound effect on her and her family’s well-being [1]. Malawi’s modern contraceptive prevalence among women of reproductive age was 45% in 2015–2016 and 30% among women at six months postpartum [2,3]. Though the country’s contraceptive prevalence rate has steadily increased over the past two decades, unmet need for family planning among married women in the Central Region, where this study was conducted, was 16% in 2016, and the total fertility rate in the region (4.4) remained higher than the total wanted fertility rate (3.4), indicating a need to identify new avenues to reach women with counseling and services [2]. 

The Government of Malawi’s *Health Sector Strategic Plan II* highlights that “opportunities shall be created to facilitate integration of health service delivery to leverage on efficiency and effectiveness in addressing health needs of the people of Malawi” [4] and identified integration of family planning with other health services as a “critical strategy in enhancing the availability of family planning services” within the *Malawi Costed Implementation Plan for Family Planning, 2016–2020* [5]. In practice, however, this integration has not been fully realized, as services have been provided in parallel with different schedules or by different providers. 

Global stakeholders have identified “ensuring that family planning counseling and services are linked to infant vaccination contacts through well-managed primary health care services” as a promising high-impact practice for family planning [6,7]. With comparatively high coverage rates, immunization services provide a favorable opportunity for family planning service integration in Malawi; nationwide three-dose diphtheria–pertussis–tetanus (DPT3) vaccination coverage, a common proxy indicator for full childhood immunization, was 93% in 2016 [2]. However, global leaders, including the United States Agency for International Development (USAID) and the United Nations Population Fund, have indicated that more evidence is needed to advance family planning and immunization service integration from a “promising” to a “proven” practice, as evidence around its effect on immunization outcomes is thin, and gaps exist in understanding optimal integration models and contextual factors that affect uptake. Additionally, most of the published evidence focuses on health facilities, while little exists on use of outreach platforms for family planning and immunization service integration.

This mixed methods process evaluation examines the implementation and results of an intervention to systematically integrate family planning and immunization services in all health facilities and associated community sites in Ntchisi and Dowa districts in the Central Region of Malawi from June 2016 to September 2017. Both Dowa and Ntchisi districts are rural and had total populations of 732,342 and 276,481, respectively, around the time of the study [8]. Health services are delivered through district hospitals and health centers, and their associated community sites, and operated by the government and the Christian Health Association of Malawi.

**Figure 1:**
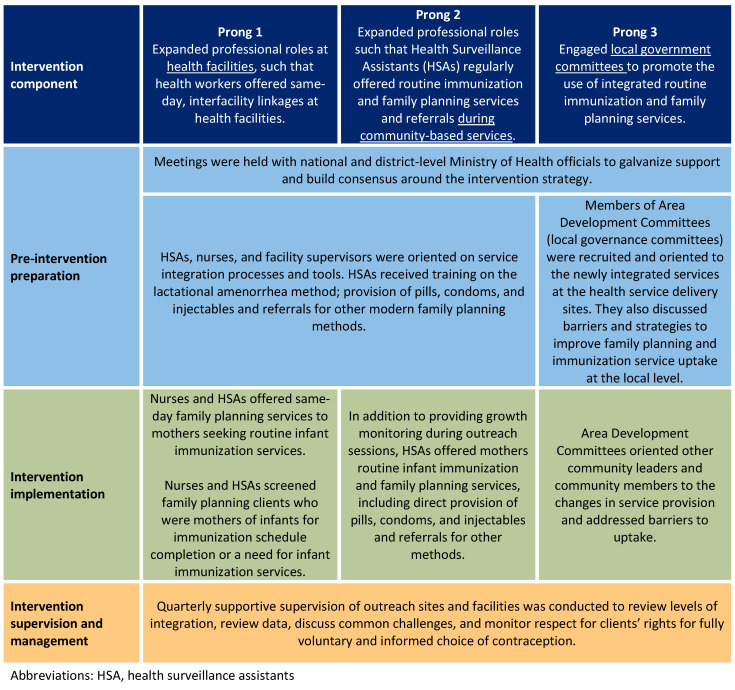
The family planning–immunization integration implementation approach.

For the purpose of this paper, “family planning and immunization integration” refers to activities that deliberately linked the two services through same-day provision of both services or referral for the other service at another site or on a different day [6]. The Ministry of Health’s approach, supported by USAID’s flagship Maternal and Child Survival Program (MCSP), included three prongs: (1) service integration at health facilities; (2) service integration at community sites; and (3) community engagement to address barriers to integrated service utilization (Figure 1). 

The intervention included building the family planning knowledge and skills of health surveillance assistants (HSAs), a cadre of paid community health workers who are linked to a primary-level health facility and provide a range of basic health services at their facility and through community outreach sessions. This work built on a successful pilot initiative implemented by the Maternal and Child Health Integrated Program and the government of Liberia in 2012, which led to substantial increases in new contraceptive users [6]. A follow-up study in Liberia running concurrently with this Malawi study examined the results of an expanded version of the previous Liberia approach scaled to additional sites, demonstrating slight increases in family planning uptake with no negative effect on immunization utilization rates [9]. Similar studies in Rwanda and Togo demonstrated positive effects on family planning outcomes, with no negative effect on immunization services [10,11]. 

This mixed methods process evaluation aimed to address the following research questions:

- Is integration of family planning and immunization services at community sites and health facilities associated with a change in experience of care and uptake of modern contraception and routine infant immunization?
- How do contextual factors affect implementation, provision of care, and use of integrated services?

## 2. Materials and Methods

### 2.1. Quantitative Data Collection and Analysis

We analyzed outcomes of interest during the 21-month study period spanning five months before the intervention (January–May 2016) and 16 months of implementation (June 2016–September 2017). Outcomes of interest included new modern family planning users (clients who are “seeking a family planning method for the first time in their lifetime” [12]), total modern family planning users (sum of new and continuing users), contraceptive method mix, first-dose diphtheria–pertussis–tetanus vaccine (DPT1) and DPT3 immunization doses administered, and the DPT1 to DPT3 dropout rate. All quantitative data analyses focused on 14 health facilities in Dowa that met the inclusion criterion of having complete reports in the national health information system for at least 17 months (80%) of the 21-month study period. The remaining 15 health facilities in Dowa and 14 health facilities in Ntchisi had substantial gaps in family planning data and thus were excluded from quantitative analyses. 

We used a fixed effects regression model to measure differences in outcomes between the calendar quarter immediately preceding implementation, March–May 2016, and the same quarter the next year, March–May 2017. Panel data were included for the 14 facilities, for a total of 70 observations over the March–May period each year. Using a fixed effects model allowed the team to control for unknown/unmeasured differences between the individual facilities at each time point. Quantitative data analysis was conducted using Microsoft Excel 2013 (Microsoft Inc., Redmond, WA, USA) and Stata/SE 15.0 (StataCorp, Lakeway, TX, USA) statistical packages.

### 2.2. Qualitative Data Collection and Analysis

Qualitative data was collected in both Dowa and Ntchisi districts during September 2017. Within each district, one hospital and three health centers (collectively referred to as “facilities”), as well as one community site per facility were purposively selected in each district. Sites included both high- and low-performing facilities based on observations during supervision of the intervention, and community sites scheduled to provide services on the day of the data collection visit at the associated facility.

At each site, semi-structured in-depth interviews (IDIs) were conducted with one family planning provider (nurse), one HSA, and one facility supervisor. Additional interviews were conducted with up to three district representatives and up to three national Ministry of Health representatives. Semi-structured focus group discussions (FGDs) were conducted in the catchment area of the health facilities selected for the qualitative study. FGD participants, purposively identified through service ledgers, included mothers and fathers of children under 1 year of age, including women who accepted and declined referrals (from family planning to immunization services or from immunization to family planning) at community sites and health facilities. Where it was not feasible to gather a sufficient number of participants for FGDs, IDIs were carried out with mothers of children under 1 year of age. 

IDIs and FGDs were audio recorded and data collectors took hard copy notes. IDIs and FGDs were transcribed verbatim in Chichewa and translated to English. Translations were reviewed by the data collection supervisor and Chichewa-speaking members of the research team. 

An analysis team led by a local consultant reviewed translated interview transcripts and identified dominant themes, which were used to develop a coding structure to guide the qualitative analysis. The coding process allowed for identification of additional themes from the interviews. After coding was completed by the Malawian team, the US-based research team examined a subset of transcripts to verify themes and confirm any additional emerging concepts. Key themes explored included: barriers and motivators for postpartum contraceptive uptake and immunization schedule completion, including associated costs; perceptions of service integration, including anticipated benefits/challenges and recommendations; client flow; service provider roles and responsibilities; commodity availability; and fidelity to the implementation strategy and intervention. Saturation of responses around these themes was reached. Qualitative analyses were conducted using hand coding and NVIVO version 11 (QSR International, Melbourne, Australia). 

### 2.3. Ethics Approval and Consent to Participate

This study was approved by Johns Hopkins University and Malawi National Health Sciences Research Committee IRBs (JHSPH IRB #00007595, Malawi NHRSC #1815).

## 3. Results

Study results are presented around the effect of service integration on service utilization (quantitative data) and on experience of care (qualitative data), as well as the contextual factors that affected implementation of service integration (qualitative data). An overview of the total respondent sample for the qualitative data is presented in Table 1. 

### 3.1. Implementation of the Intervention

Routine immunization and family planning services were integrated in all 43 health facilities and 373 associated community sites across Dowa and Ntchisi, and 306 HSAs were trained to offer counseling; direct provision of condoms, pills, and injectables; and referrals for long-term and permanent methods. Meetings were convened with local government committees to discuss and identify strategies to address barriers to use of family planning and immunization services.

### 3.2. Effect of Integration on Service Delivery

#### 3.2.1. Family Planning

**Figure 2:**
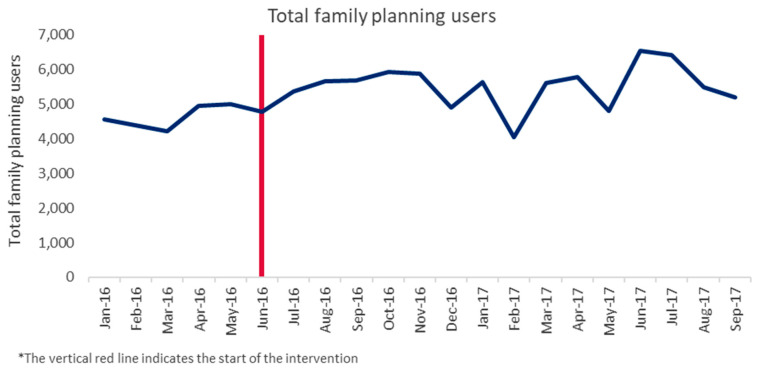
Total family planning users at implementation sites in Dowa, pre- and postimplementation.

Analyses revealed increases in contraceptive use in Dowa district (Figure 2). 

At the subset of 14 facilities and their associated community sites in Dowa, the total number of women accessing family planning services during the study period increased by 14%, from an average of 4722 total family planning users per month (both new and returning users) during the pre-implementation period (March–May 2016) to 5404 clients during the same period a year later (March–May 2017), although increasing trends in contraceptive use had begun prior to the start of the intervention.

The fixed effects regression model showed significant (*p* ≤ 0.001) increases in total family planning users between similar pre- and postintervention periods in 2016 and 2017 (Table 2), but did not find a statistically significant increase in new users (*p* = 0.591). The program’s effect on total users appears to be driven by significant increases in both new (*p* ≤ 0.001) and total (*p* ≤ 0.001) family planning users accessing community-based services between the pre- and postintervention periods (Table 2).

**Figure 3:**
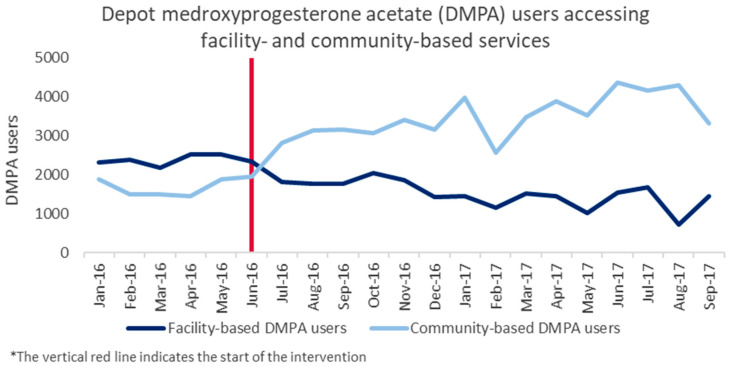
Facility- and community-based depot medroxyprogesterone acetate (DMPA) provision, pre- and postimplementation in Dowa.

The proportion of all users who accessed family planning through community-based platforms increased following the start of the program. From March to May 2016 (prior to the intervention), 37.8% of the total family planning clients and 28.1% of the new family planning clients accessed community-based services. These percentages increased to 69.1% and 53.0%, respectively, by March–May 2017. Community-based services overtook facility-based services as the more commonly accessed service delivery platform in June–July 2016 for total family planning clients, and in August–September for new family planning clients. As seen in Figure 3, these shifts were largely driven by changes in the place of depot medroxyprogesterone acetate (DMPA) provision, which shifted from being primarily facility-based to primarily community-based in June–July 2016. 

**Figure 4:**
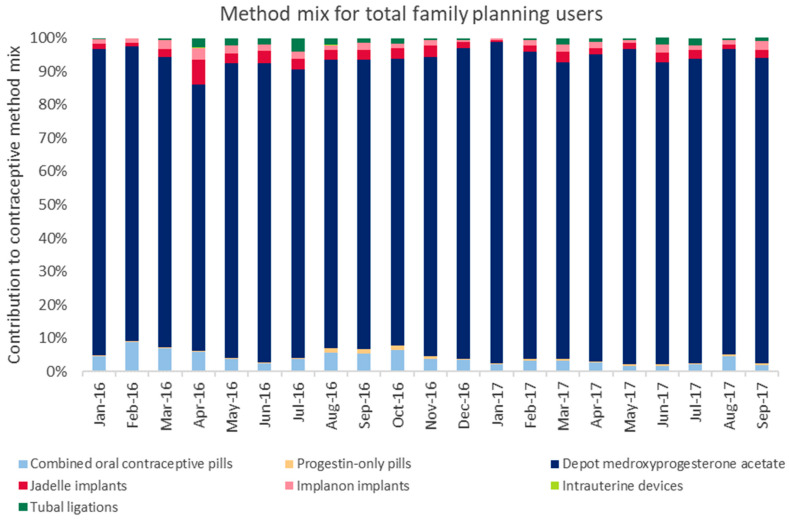
Method mix for total family planning users, pre- and postimplementation in Dowa.

There was no substantive change in the overall method mix for either the total or new family planning clients (Figure 4). 

DMPA was the most common method used among all family planning clients throughout the 21-month study period (January 2016–September 2017), including the pre- and postintervention periods. On average, DMPA represented 89.7% of the method mix among total family planning clients, and 65.3% of the method mix for new family planning clients. Short-acting methods (combination oral contraceptive pills, progestin-only pills, and DMPA) constituted 94.2% of the method mix for total family planning clients and 71.2% of the method mix for new family planning clients. Among total family planning clients, implants (Implanon and Jadelle) were the most common long-acting contraceptive, which constituted 4.4% of the method mix, on average. Similarly, Jadelle was the most common long-acting contraceptive for new family planning clients, making up 10.8% of the method mix for new users (not shown), followed by tubal ligation (9.8%) and Implanon (8.5%). 

#### 3.2.2. Immunization

Within the same subset of 14 facilities in Dowa, provision of DPT1 and DPT3 stayed relatively consistent throughout the 21-month study period. The average number of DPT1 doses administered was 862 during March–May 2016 (preintervention) and 901 during March–May 2017 (postintervention). The average numbers of doses of DPT3 administered for the same time periods were 824 and 949, respectively. The regression models comparing DPT1 and DPT3 doses administered during pre- and postintervention periods only found a significant increase in the number of DPT3 doses administered (*p* = 0.003). However, both immunization models had high interclass correlation values (Table 2), suggesting that much of the differences over time were likely due to variations between facilities themselves.

During the postintervention period (June 2016–September 2017), the DPT1 to DPT3 dropout rate remained under 10% except for one month (January 2017) when it rose to 13%. The dropout rate across the 14 facilities averaged 4% for March–May 2016 and −5% from March–May 2017.

Over the 21 months under study, minimal fluctuations were observed in the percentage of total doses of DPT1 or DPT3 provided through community-based services as opposed to facilities. The percent of doses provided by community-based services ranged from 55% to 73% for DPT1 (mean of 66% during the pre- and postintervention periods) and 62% to 71% for DPT3 (mean of 70% preintervention and 68% during the postintervention period). 

**Figure 5:**
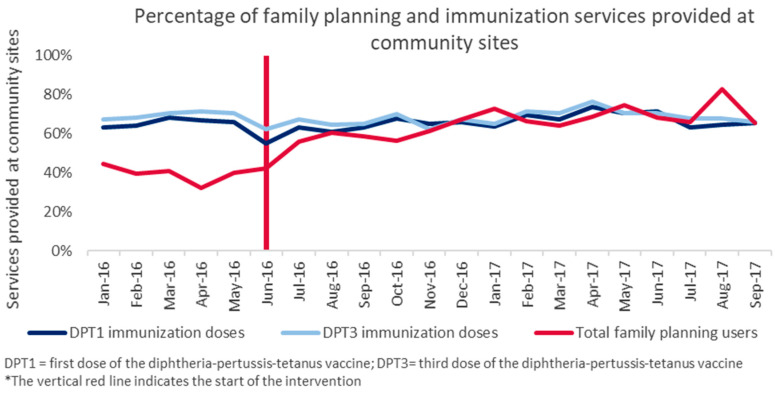
Percentage contraceptive methods and immunization doses administered at the community level in Dowa.

Unlike family planning, the proportion of immunization clients accessing community-based services did not change over the postintervention period. The proportion of total women receiving family planning from community-based services increased to about the same as infants receiving DPT1 and DPT3 at community sites, with all three services remaining between 63% and 83% in the final six months of the study (Figure 5).

### 3.3. Effect of Integration on Experience of Care

#### 3.3.1. Benefits Perceived by Mothers and Fathers

Mothers and fathers described how service integration affected their experiences of care. Benefits observed included time savings, geographic convenience, and improved knowledge and understanding of the services. Respondents stated that service integration saved them time because they were able to receive two services in one trip to a health facility or community outreach session. Women also appreciated the opportunity to learn about the other service when they had originally sought out only one of the two. 

An immunization referral acceptor in Dowa said:

There are some benefits because the baby gets immunized at the right time and I would also get my family planning at a good time, making me a happy and proud mum that my baby is growing healthy and I will be able to do other house chores.

One father in Ntchisi mentioned:

This is very good because it has reduced the time the mothers were wasting instead of doing household chores due to coming different days for immunization and family planning services…. The health facility is also quite far in this area about 6 km so this integration is helping so the mother does not have to walk this distance twice to access the care. The men also escort the women to the hospital for these services, so the integration is also giving us [men] time to do some businesses and going the field to farm.

Referral acceptors widely indicated that they felt they had a choice of whether to accept the referral and did not feel forced by service providers.

#### 3.3.2. Challenges Perceived by Mothers and Fathers

With regard to experience of care, the main challenge mothers mentioned was long waiting times at community sites due to delayed service provider arrival and the large number of clients to be served. A family planning referral refuser in Ntchisi noted:

Immunization comes from very far… [and] comes here late so we end up leaving this place very late. Our friends who are not immunizing their babies that day are long gone while we are still busy with immunization.

The time allotted for service provision, particularly at community sites, was also perceived to be short, leading some to be turned away. For example, a family planning referral acceptor in Dowa reported:

If we delay just by a little time we are told to go back home and come the next month.

#### 3.3.3. Provider Perspectives

Nurses and HSAs observed that service integration had improved their provision of health services. They reported that before the integrated services, providers only concentrated on their respective fields without much attention to other services. Now they were able to discuss both services with clients. As a family planning provider in Dowa mentioned:

… *before there was no integration; everyone was just concentrating on what they are doing. If it’s about family planning we would just assist the woman on family planning with no concern on the child; same way for those doing growth monitoring in the children, there would be no concern if she is doing family planning, but now they access all services so they save time and have no excuse.*

Almost all health workers also observed that the introduction of integrated family planning and immunization services greatly improved referrals of clients between the two services. For example, some facilities ensured that HSAs escorted women from the immunization clinic to the family planning clinic; other facilities ensured that women were given a referral note. 

The main challenges for service integration mentioned by providers included increased workload and documentation. As a result of this, health workers observed that clients had to wait for additional time to be seen and providers sometimes forgot to complete required documentation. As a supervisor/manager in Dowa noted:

The workload increases; we are few health workers here. Another thing is that people are kept waiting for longer, if you are alone then you have to give family planning and then you should take them for immunization, and search for someone who has to help them with that if you find that there is no one there.

### 3.4. Contextual Factors Affecting Implementation and Integration Outcomes

The service integration approach took different forms from site to site, with adaptations made depending on facility level, staffing, and other factors. While many clients said that integrated services were available at their facility or community site, some respondents indicated that mothers at immunization services did not receive family planning information, and that the services were still being provided on separate days. Respondents also noted that the link between HSAs and nurses (between community sites and health facilities and within health facilities) was not as strong as anticipated because referrals were not consistently provided. From the qualitative data, contextual factors affecting integration outcomes included commodity availability, community linkages, family planning concerns and norms, and partner engagement. 

#### 3.4.1. Commodity Availability

While program managers generally did not express concern about the availability of family planning and immunization commodities, some providers observed occasional stock-outs of contraceptives and vaccines, which presented a challenge for the implementation of integrated services and potential for increased demand. The immunization services faced additional challenges with maintenance of cold chain at the point of service delivery due to malfunctioning refrigerators. 

#### 3.4.2. Community Linkages

Respondents generally expressed that the program had involved community leaders well, but in order to address remaining barriers to service uptake, there was need to expand involvement to include other types of community stakeholders. As noted by a supervisor/manager in Ntchisi:

*To increase community sensitization to take a leading role we already started meeting with GVHs [group village headmen/women] to help us with sensitizing the community so that there should be uptake of services so that the community should know that when they go for vaccination service they can also receive family planning services*…

#### 3.4.3. Family Planning Concerns and Norms

One of the main barriers to accessing family planning services was concerns about negative health effects of contraceptive methods and social norms opposing family planning. As noted by a family planning provider in Ntchisi:

*If they hear rumors and have some fears about family planning, some women may not access the services*… *for example, people say that when you are taking family planning methods, it means you will never have a child, so there is need to dispel those rumors. If you counsel the person properly, she understands.*

#### 3.4.4. Partner Engagement and Male Involvement

Some health workers reported that men restricted their wives from accessing family planning services. For example, an HSA in Ntchisi noted:

In my view, I think the problems are coming due to lack of male involvement; there are some men that are restricting their wives from accessing family planning services so women have devised to use two health passports [client-held health records] where one health passport is strictly for family planning services; in that way the husband does not know that the wife is on family planning.

## 4. Discussion

This study sought to understand whether integration of family planning and immunization services at community sites and health facilities was associated with a change in experience of care and uptake of modern contraception and routine infant immunization services, as well as how contextual factors affect implementation, provision of care, and use of integrated services. 

This study’s finding that family planning integration had no statistically significant effect on immunization outcomes (consistent with results from studies in Liberia [7,9], Rwanda [10] and Togo [11]) should be reassuring to the immunization community in that service integration was not shown to detract from immunization outcomes. DPT dropout rates remained under 10% during the study period, except for one month (likely unrelated to the intervention). According to the World Health Organization, a dropout rate of 10% or higher is indicative of problems with service utilization [13]. We did not expect that the intervention would affect uptake of DPT1, which can be used as a proxy for immunization access. The finding that the number of DPT3 immunization doses administered and the proportion of DPT1 and DPT3 immunization doses provided at outreach were not negatively impacted by the intervention suggests parents were comfortable receiving family planning services when bringing their infants for immunization. 

Statistically significant increases in the total number of family planning clients at the 14 facilities in Dowa with at least 80% of monthly reports available, are likely, in part, a result of other previous and ongoing projects working to improve family planning uptake across the country and district. As such, while this service integration effort was not solely responsible for the total increase in family planning clients throughout the study period, we believe that it contributed to it. 

The lack of statistical significance among new contraceptive users may be due to the relatively small numbers of family planning clients classified as new users and that “lapsed” postpartum users (who had previously used a family planning method) are not considered new users. At a global level, experts have identified challenges with the “new user” indicator [14].

The most notable change during the study period was in the proportion of women accessing family planning at community sites. The average number of family planning clients who accessed community-based services almost doubled between the pre- and postintervention period, while the number of clients accessing services at facilities fell by roughly 30%. The proportion of total family planning clients accessing community-based services increased to an average of 64.4% after implementation of the intervention, which was close to the percentage of infants receiving DPT1 and DPT3 from community-based immunization services; 65.6% and 67.8%, respectively. There was a similarly significant increase in the number of new family planning users accessing services at community sites, but not a concurrent significant decrease in the number accessing facility-based services. This suggests that many women, and continuing family planning users in particular, may switch from facility-based to community-based family planning services because the latter are more accessible, while 30–40% of women may prefer (or have easier access to) facility-based services. Other recent studies have reached similar conclusions. A discrete choice experiment in rural Malawi found that improving the quality of community-based services shows more potential for expanding young (aged 15–24) people’s access to family planning services in rural areas than facility-based services and that service improvement may be an important tool for increasing the uptake of sexual and reproductive health services in this population [14]. 

Accessibility emerged as a major perceived benefit of the service integration, with respondents appreciating not having to travel as far to reach community-based services and not having to travel on two different days. Self-injection of injectable contraceptives was demonstrated to be feasible in Malawi [15], and the outreach platform can serve as an opportunity for initiation and follow-up of those users, giving women a full year on the method in only one visit. Training HSAs in provision of Implanon NXT (contraceptive implants) could also offer an opportunity to expand contraceptive options available through the outreach platform [16]. Attention should be paid to ensuring that HSAs counsel on the full range of contraceptive options, provide referrals, and facilitate women’s use of long-acting and permanent methods, as desired, especially considering the low representation of long-acting reversible contraceptives among the method mix in this study. 

Results from this study must be interpreted within the context of Malawi’s comparatively high contraceptive prevalence and projections for contraceptive growth in the country. Malawi is classified as “Stage 3” along the S-curve for contraceptive growth, indicating high contraceptive prevalence with growth expected to slow down and eventually stop as contraceptive prevalence reaches its maximum point [3]. As noted by Track20;

*Programs at this stage need to focus on long-term sustainability, continued improvements in service quality, and expanding the range of methods available. At this stage, rather than focusing on further growth, goals and objectives should be focused on equity indicators and government financial commitments*.

[3]

The focus on equity includes identifying pockets of users with unmet need, including postpartum women. While this study shows that service integration is one strategy for increasing contraceptive uptake, stakeholders in Malawi will need to consider what barriers continue to prevent additional users, beyond convenience and access. 

One notable factor that emerged was the inconsistent implementation of the approach throughout the two districts. Future efforts should take an intentional change management approach for improving implementation fidelity (and adaptation as needed) through routine monitoring and supervision, especially with a move toward expanded scale of implementation.

Because implementation was conducted at full saturation (all facilities and associated outreach sites) in two districts, we were able to generate insights regarding scale-up. These insights include the importance of applying a health systems approach that includes engaging district immunization and family planning representatives in rollout and supervision, ensuring adequate human resources and commodities are available, clarifying the scope of work for the cadres involved, and building the capacity of data capture and reporting systems to monitor progress. 

There are specific limitations of the study that should be noted. One of the main limitations of this study was the quality of referral and family planning data. Gaps in reporting of monthly family planning service statistics at all facilities in Ntchisi and fifteen of 29 facilities in Dowa prevented comparison of outcomes between the two districts and may have biased the results. Women in the extended postpartum period were the primary focus of the intervention; however, we were unable to generate a clear picture of the effect on postpartum contraceptive uptake, as postpartum family planning indicators (e.g., counseling on postpartum contraceptive options, women receiving a contraceptive method before discharge from a facility after childbirth, women receiving a contraceptive method during the immediate or extended postpartum period, lactational amenorrhea method use) are not tracked in the national health management information system (HMIS). Due to this limitation, it is likely that the effect of the intervention on postpartum contraceptive use was under-represented in the new and total family planning user numbers presented in this paper. Future efforts could explore how to include simple referral tracking within national tools, including tracking the postpartum status of family planning clients. Use of mobile phones for family planning data collection has demonstrated promise at similar sites in Malawi and should be further considered [17]. 

Though it was the most complete data set, immunization data collected in District Vaccine Data Management Tool often underrepresents the actual doses of DPT1 and DPT3 administered each month and thus might not accurately reflect outcomes [18]. Data were only analyzed for one year after the implementation rollout, and thus may not provide a full picture of seasonal changes from year-to-year. Finally, due to the observational study design employed, it was not possible to analyze and describe results, such as the increase in total family planning users, that were attributable to the specific integration of family planning and immunization services, efforts to strengthen community-based services, other concurrent programs, or existing secular and temporal trends. Nevertheless, this study makes an important contribution to the existing family planning and immunization integration evidence base around the effect on immunization services, considerations for scale-up, quality of care, integration models, and the role of contextual factors. The study’s results complement findings of the concurrent study in Liberia, which also highlighted the importance of contextual factors and evaluation design considerations [9].

## 5. Conclusions

Integration of family planning and immunization services was perceived to be feasible and beneficial by clients and providers, indicating a win-win for both services. The intervention appears to have facilitated continued increases in total contraceptive use and accessibility and improved perceptions regarding quality of care, which are important achievements in a country with already comparatively high modern contraceptive prevalence. In spite of these positive findings, barriers to family planning uptake and postpartum family planning measurement remain. The Malawi government and other stakeholders are pursuing other efforts to integrate services, and it is hoped that this study feeds into ongoing decision-making regarding health systems strengthening and strategies for reducing missed opportunities for care for women, children, and their families in Malawi and beyond.

## Figures and Tables

**Table 1:** Respondent characteristics.

Data Collection Activity	Participant Type	Number of Participants: Dowa	Number of Participants: Ntchisi	Number of Participants: Total (Dowa + Ntchisi)
**Focus group discussions**	Men with infants < 1 year	1 FGD (7 respondents)	1 FGD (6 respondents)	2 FGD (13 respondents)
	Women with infants < 1 year who accepted immunization referrals	4 FGDs (36 respondents)	4 FGDs (23 respondents)	8 FGDs (59 respondents)
	Women with infants < 1 year who accepted FP referrals	8 FGDs (48 respondents)	8 FGDs (64 respondents)	16 FGDs (112 respondents)
	Women with infants < 1 year who declined FP referrals	8 FGDs (37 respondents)	3 FGDs (20 respondents)	11 FGDs (57 respondents)
**In-depth** **interviews**	Women with infants < 1 year who declined FP referrals	0	3	3
	Community-based HSAs	2	2	4
	Facility-based HSAs	4	4	8
	FP providers (nurses)	4	4	8
	Facility supervisors	4	3	7
	Program managers: district	3	3	6
	Program managers: national	2		

FGD = focus group discussions; FP = family planning; HSAs = health surveillance assistants.

**Table 2:** Effect of intervention on use of family planning and immunization services; Dowa district, March–May 2016 vs. March–May 2017.

Indicator	Coefficient *	Standard Error	95% Confidence Interval	Rho
New community-based FP users	12.0	2.43	7.12–16.86	0.328
Total community-based FP users	186.6	23.86	138.8–234.4	0.350
New facility-based FP users	−9.0	5.3	−19.6–1.5	0.376
Total facility-based FP users	−63.3	16.3	−95.9–−30.7	0.512
All new FP users	2.9	5.4	−8.9–13.9	0.390
All FP users	123.3	24.5	74.2–172.4	0.571
DPT1 doses	3.1	2.6	−2.1–8.2	0.875
DPT3 doses	9.2	2.9	3.4–15.0	0.810

FP = family planning; DPT = diphtheria–tetanus–pertussis. * Fixed effects panel regression models were used to estimate coefficients for each indicator. Data were grouped by facility and month.
